# Effect of Deworming on Physical Fitness of School-Aged Children in Yunnan, China: A Double-Blind, Randomized, Placebo-Controlled Trial

**DOI:** 10.1371/journal.pntd.0002983

**Published:** 2014-07-10

**Authors:** Peiling Yap, Fang-Wei Wu, Zun-Wei Du, Jan Hattendorf, Ran Chen, Jin-Yong Jiang, Susi Kriemler, Stefanie J. Krauth, Xiao-Nong Zhou, Jürg Utzinger, Peter Steinmann

**Affiliations:** 1 Department of Epidemiology and Public Health, Swiss Tropical and Public Health Institute, Basel, Switzerland; 2 University of Basel, Basel, Switzerland; 3 Helminthiasis Division, Yunnan Institute of Parasitic Diseases, Pu'er, People's Republic of China; 4 Menghai Center for Diseases Control and Prevention, Menghai, People's Republic of China; 5 Institute of Social and Preventive Medicine, University of Zurich, Zürich, Switzerland; 6 Centre Suisse de Recherches Scientifiques en Côte d'Ivoire, Abidjan, Côte d'Ivoire; 7 National Institute of Parasitic Diseases, Chinese Center for Diseases Control and Prevention, Shanghai, People's Republic of China; Rosetta Genomics, Israel

## Abstract

**Background:**

There is considerable debate on the health impacts of soil-transmitted helminth infections. We assessed effects of deworming on physical fitness and strength of children in an area in Yunnan, People's Republic of China, where soil-transmitted helminthiasis is highly endemic.

**Methodology:**

The double-blind, randomized, placebo-controlled trial was conducted between October 2011 and May 2012. Children, aged 9–12 years, were treated with either triple-dose albendazole or placebo, and monitored for 6 months post-treatment. The Kato-Katz and Baermann techniques were used for the diagnosis of soil-transmitted helminth infections. Physical fitness was assessed with a 20-m shuttle run test, where the maximum aerobic capacity within 1 min of exhaustive exercise (VO_2_ max estimate) and the number of 20-m laps completed were recorded. Physical strength was determined with grip strength and standing broad jump tests. Body height and weight, the sum of skinfolds, and hemoglobin levels were recorded as secondary outcomes.

**Principal Findings:**

Children receiving triple-dose albendazole scored slightly higher in the primary and secondary outcomes than placebo recipients, but the difference lacked statistical significance. *Trichuris trichiura*-infected children had 1.6 ml kg^−1^ min^−1^ (*P* = 0.02) less increase in their VO_2_ max estimate and completed 4.6 (*P* = 0.04) fewer 20-m laps than at baseline compared to non-infected peers. Similar trends were detected in the VO_2_ max estimate and grip strength of children infected with hookworm and *Ascaris lumbricoides*, respectively. In addition, the increase in the VO_2_ max estimate from baseline was consistently higher in children with low-intensity *T. trichiura* and hookworm infections than in their peers with high-intensity infections of all soil-transmitted helminths (range: 1.9–2.1 ml kg^−1^ min^−1^; all *P*<0.05).

**Conclusions/Significance:**

We found no strong evidence for significant improvements in physical fitness and anthropometric indicators due to deworming over a 6-month follow-up period. However, the negative effect of *T. trichiura* infections on physical fitness warrants further investigation.

## Author Summary

Children from the developing world are often burdened with intestinal worms due to poor water supply, sanitation, and hygiene. However, the assessment of the burden due to intestinal worms is difficult, and thus, the benefits of deworming are unclear. In this study, we determined the effect of deworming on the physical fitness and strength of 9- to 12-year-old children in Yunnan, China, where intestinal worms are common. Children were treated with triple-dose albendazole or placebo and monitored over a 6-month period. Stool samples were collected for the diagnosis of intestinal worm infections. Physical fitness was estimated with a 20-m shuttle run test and physical strength was assessed with grip strength and standing broad jump tests. Children receiving triple-dose albendazole scored slightly higher values in the primary and secondary outcomes than those children who were given placebo. However, the differences were not significant. We also found that children infected with intestinal worms performed significantly worse in the physical fitness and strength tests than their non-infected counterparts. In particular, the negative impact of whipworm infection on physical fitness warrants further investigation.

## Introduction

Soil-transmitted helminths, namely *Ascaris lumbricoides*, *Trichuris trichiura*, and the hookworms (*Ancylostoma duodenale* and *Necator americanus*), are the most common parasitic worm infections of humans. Indeed, more than 1 billion people are infected and approximately 5.4 billion people are at risk of infection [1]–[3]. In 2011, an estimated 875 million children, 70% of whom were school-aged, were at risk globally [4]. Impoverished communities with poor hygiene and no access to clean water and improved sanitation are especially vulnerable [5], [6].

The global burden of soil-transmitted helminthiasis is currently estimated at 5.2 million disability-adjusted life years (DALYs), mainly due to sub-clinical morbidities, but also anemia and reduced cognitive and physical development [7]–[9]. Infections are largely chronic and usually asymptomatic, and hence the study and quantification of the morbidity associated with soil-transmitted helminth infections are difficult, and only few studies have ventured to do so. In particular, no conclusive evidence has yet been established whether reduced physical fitness or strength are a consequence of soil-transmitted helminth infections. Physical fitness has been positively correlated with academic performance through enhanced memory and attention [10], [11], while physical strength is demanded in labor-intensive agriculture jobs, which often provide the main source of income in rural communities of the developing world [12]. A lack in both attributes due to soil-transmitted helminthiasis could arguably prevent school-aged children living in impoverished conditions from realizing their full potential and perpetuate their entrapment in the vicious cycle of poverty and poor health [13], [14].

Based on the rationale that lowering infection intensity would help to control morbidity associated with chronic helminth infection, and that morbidity is infection intensity-dependent, the World Health Organization (WHO) advocates periodic deworming of at-risk populations (e.g., school-aged children and pregnant women) with single-dose albendazole (400 mg) or mebendazole (500 mg) [15], [16]. Such an approach indeed reduces infection intensity in the target population, but high-quality evidence on the health benefits of de-worming in children is scant [17], [18]. Two randomized controlled trials have shown that physical fitness in school boys infected with soil-transmitted helminths improved 7 weeks to 4 months after treatment with single-dose albendazole [19], [20]. Physical fitness was also negatively correlated with *T. trichiura* and hookworm infections in two cross-sectional studies [21], [22] but another cross-sectional study did not find any correlation between physical fitness and soil-transmitted helminth infections [23]. However, it is important to note that in the latter study, both the prevalence and intensity of soil-transmitted helminth infections were very low.

We designed a randomized controlled trial to investigate the health benefits of deworming and thereby deepen our understanding of the burden caused by soil-transmitted helminth infection among school-aged children. The study was conducted in a highly endemic area in the People's Republic of China (P.R. China) and assessed the effects of triple-dose albendazole on physical fitness and strength of initially soil-transmitted helminth-infected children. The infection and fitness dynamics were then studied over a 6-month period post-treatment. Changes in anthropometric indicators and hemoglobin levels were also measured, and are reported as secondary outcomes.

## Methods

### Ethics Statement

The study protocol was approved by the institutional research commission of the Swiss Tropical and Public Health Institute (Basel, Switzerland). The ethics committee of Basel (EKBB, reference no. 144/11) and the Academic Board of the National Institute of Parasitic Diseases, Chinese Center for Disease Control and Prevention (Shanghai, P.R. China) provided ethical clearance. The trial is registered with Current Controlled Trials (identifier: ISRCTN 25371788).

The village doctor, chief, and teachers of each village were briefed on the aims of the study. With help from the teachers, the investigators further explained the procedures to the children and their parents/guardians. Written informed consent was obtained from parents/guardians, whereas children assented orally. Data were kept anonymous. After the 6-month final follow-up, all children attending the five schools were given triple-dose albendazole (3×400 mg) irrespective of their infection status, study participation, and treatment during the study. Children diagnosed with *Strongyloides stercoralis* were offered a single dose of ivermectin (200 µg/kg).

### Participants

Participants were recruited from five primary schools, where a 70% or higher prevalence of soil-transmitted helminth infections had been detected during a rapid appraisal. All schools belonged to villages exclusively inhabited by the Bulang ethnic minority group, and were located in the mountainous Bulangshan township bordering Myanmar, a sub-division of Menghai county in Xishuangbanna Dai autonomous prefecture, situated in Yunnan province, P.R. China. The five villages are: (i) Sandui (geographical coordinates: 21°33′07″N latitude, 100°19′34″E longitude, altitude: 1,566 m above sea level (asl)); (ii) Kongkan (21°32′34″N, 100°20′25″E, 1,195 m asl); (iii) Laozhai (21°31′37″N, 100°18′01″E, 1,399 m asl); (iv) Laonandong (21°33′28″N, 100°21′45″E, 1,188 m asl); and (v) Mannuo (21°33′27″N, 100°23′53″E, 1,352 m asl). Prior to the current trial, no survey or control activities targeting soil-transmitted helminthiasis had been implemented in the study villages. Detailed information on the study area has been published along with data on soil-transmitted helminth re-infection patterns among participants [24]. Moreover, the epidemiology and control of soil-transmitted helminthiasis in comparable Bulang communities previously studied by our group have been described elsewhere [25], [26].

### Study Design

The study was designed as a double-blind, randomized, placebo-controlled trial with three follow-ups, and was carried out between October 2011 and May 2012. Assuming a prevalence of 70% with any soil-transmitted helminth infection and 50% loss to follow-up, the trial aimed to enroll 250 children at baseline to achieve a power of 80% at an alpha error of 5% for the detection of a 2.5 ml kg^−1^ min^−1^ difference in the maximum aerobic capacity within 1 min of exhaustive exercise (VO_2_ max estimate) between the intervention and placebo groups.

Inclusion criteria for the trial were: (i) provision of two stool samples at baseline; (ii) presence of at least one type of soil-transmitted helminth infection; (iii) no deworming treatment within 6 months before the current study; (iv) no known allergy to albendazole; (v) no major systemic illnesses as determined by a medical doctor; (vi) no concurrent participation in other clinical trials; (vii) residency in the study area for at least 1 year before enrolment; and (viii) participant should be between the age of 9 and 12 years.

**Figure 1:**
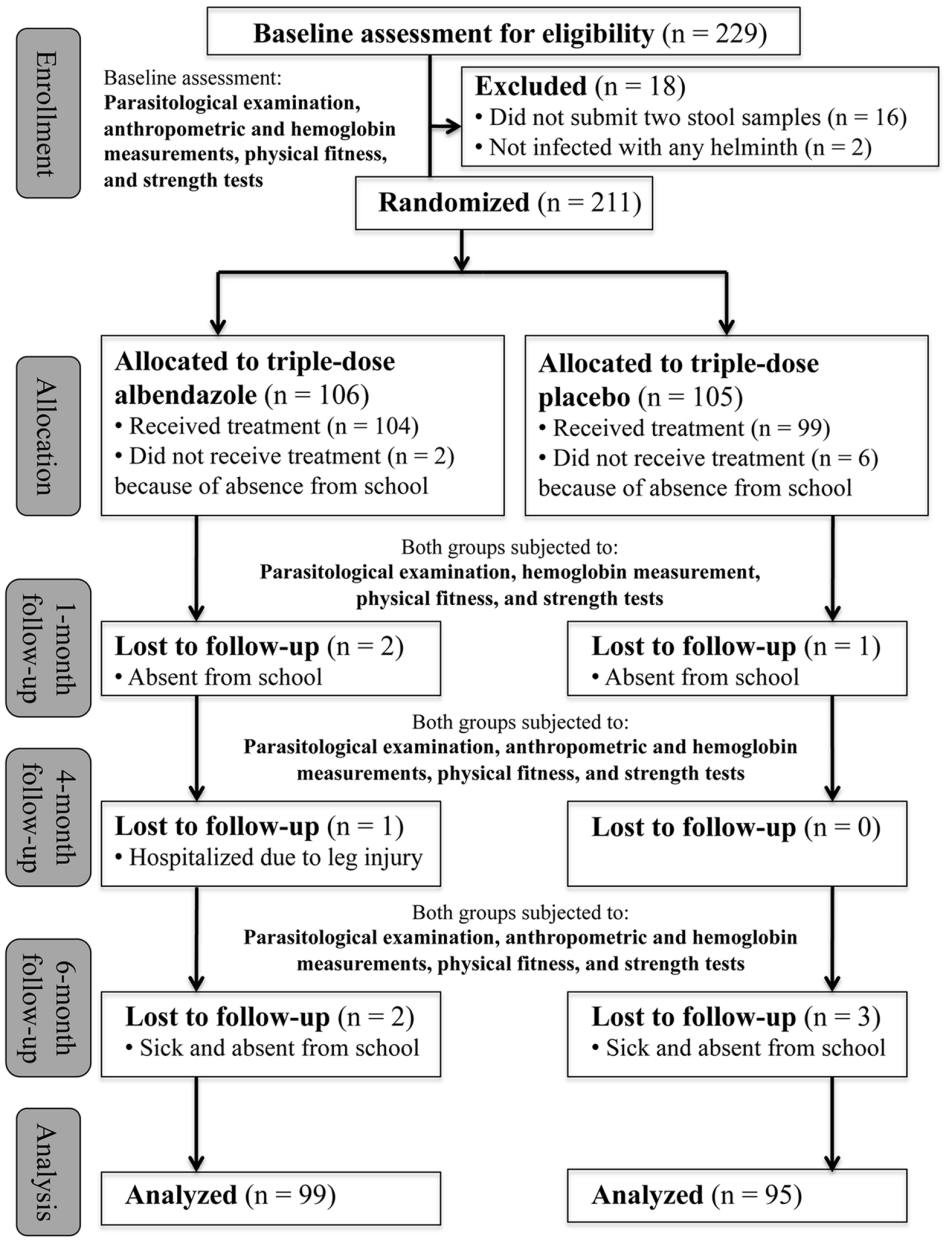
Profile of randomized controlled trial conducted in south-west Yunnan province, P.R. China, from October 2011 to May 2012.

Children aged 9–12 years who met the inclusion criteria were enrolled by field investigators for a baseline assessment involving parasitological examination, physical fitness and strength tests, and anthropometric and hemoglobin measurements. The same measurements were repeated 1, 4, and 6 months after treatment, with the exception of anthropometric indicators that were only re-assessed at the 4- and 6-month follow-ups (Figure 1).

The treatment allocation sequence was generated by a statistician using block randomization with randomly varying block sizes of 2, 4, and 6. Albendazole and placebo tablets were packaged by staff not involved in the field work into sealed envelopes marked with unique identifiers. Following the order of the class list provided by the teachers, each child was sequentially assigned a random number, which corresponded to a number on the sealed envelopes. Both children and field investigators were blinded to the nature of the tablets. The assigned triple-dose treatment (i.e., 3×400 mg albendazole (GlaxoSmithKline; London, United Kingdom) or 3× shape- and color-matched placebo (Fagron; Barsbüttel, Germany)), was started on treatment day 1 with a single dose, with subsequent doses administered every day until treatment day 3. The field investigators directly observed the consumption of each treatment by all children.

### Field and Laboratory Procedures

Two stool samples were collected from each child on consecutive days. Both the Kato-Katz (duplicate slides per sample) and Baermann techniques (one examination per sample) were used; Kato-Katz for the detection of eggs of *A. lumbricoides*, hookworm, and *T. trichiura*, and Baermann for larvae of *S. stercoralis* [27]. Additionally, stool samples were visually inspected for *Taenia* spp. proglottids. Using the Kato-Katz technique, eggs of *A. lumbricoides*, hookworm, and *T. trichiura* were counted separately and the results of both slides averaged. The mean was then multiplied by a factor of 24 to obtain the number of eggs per 1 g of stool (EPG). For quality control purposes, the two Kato-Katz slides were examined independently and results compared. Slides were re-read if inconsistencies were detected. An inconsistency was defined as a difference in the infection intensity (EPG) groupings based on WHO guidelines [15].

Physical fitness was assessed with a 20-m shuttle run test [22]. The running speed from the last completed 20-m lap and the total number of intervals completed were recorded. The child's age and speed were then converted into a VO_2_ max estimate (to the nearest 0.1 ml kg^−1^ min^−1^) with an equation put forth by Léger *et al.* [28].

Physical strength was assessed with a grip strength and a standing broad jump test. For the grip strength test, the hand span (distance from the tip of the thumb to the tip of the little finger) of the child's dominant hand was measured (to the nearest 0.5 cm) and an electronic dynamometer (Yi Lian Medicine; Shanghai, P.R. China) adjusted accordingly to provide the optimal grip span [29]. Children were asked to stand straight yet relaxed, and grip the dynamometer with the dominant hand as hard as possible for 5 sec, with the arm fully extended and without other parts of the body touching it. Each child had two tries (with a 15-sec rest in between), but only the maximum reading was recorded, to the nearest 0.1 kg. For the standing broad jump test, each child, standing behind a straight line, had two tries (with a 15-sec rest in between) to jump as far forward as possible with both legs. The jumps were recorded to the nearest 1 cm and the longer jump considered for an individual. The distance of the jump was measured from the starting line to the heel of the most back foot.

For the measurement of body height and weight, children were asked to take off their shoes and sweater before standing on a digital weighing scale (Model RCS-150; Nantong Xineng Ltd., Jiangsu, P.R. China) or stadiometer (Nantong Xineng Ltd., Jiangsu, P.R. China) [22]. Both height and weight were recorded twice, to the nearest 0.1 cm or kg, respectively, and averaged. The body mass index (BMI) was defined as (weight in kg)/(height in m)^2^; the BMI-for-age Z score (BAZ) and height-for-age Z score (HAZ) were used as indicators for wasting and stunting, respectively [30]. The thickness of skinfolds was measured at two sites, namely triceps and subscapular, with the Holtain skinfold caliper (Holtain Ltd.; Crymych, United Kingdom) [31]. Measurements were performed in triplicate to the nearest 1 mm and averaged. The sum of the mean skinfolds at both sites was used as an estimate for body fat. The hemoglobin level was measured once, to the nearest 1 g l^−1^, with a HemoCue Hb 301 system (HemoCue AB.; Ängelholm, Sweden) using a drop of blood from the ear lobe. Anemia was defined according to WHO age- and sex-specific cut-offs [32].

The socioeconomic status of the participants at baseline was assessed through a questionnaire asking the children for the education level of their parents and the main source of their household income. The full trial protocol is available as supporting information (Protocol S1).

### Statistical Analysis

Data were entered into Excel version 2008 (Microsoft Corp.; Redmond, United States of America), double-checked, and merged into a single database for statistical analysis with STATA version 10.0 (STATA Corp.; College Station, United States of America). The randomization code was broken after data entry and a series of internal consistency checks were completed. A per-protocol analysis was carried out in an un-blinded manner.

In the primary analysis, physical fitness and strength scores, anthropometric measurements, and hemoglobin levels were expressed as means, and changes in the means between baseline and treatment follow-ups were compared between treatment groups in a multivariate linear regression model. In a sub-analysis, changes in the means of physical fitness and strength indicators between baseline and follow-up were compared among children of distinct soil-transmitted helminth infection status regardless of treatment status. To further explore the effect of infection intensity on these measurements, distinct groups of children were identified using principal component and cluster analysis, based on species-specific soil-transmitted helminth log transformed egg counts at baseline and at the 1- and 4-month follow-ups. Changes in the means of physical fitness and strength indicators between baseline and follow-up were compared among six biologically meaningful groups of varying soil-transmitted helminth infection intensity.

## Results

### Baseline Characteristics

As illustrated in Figure 1, an overall compliance of 92% was achieved with only nine children lost to follow-up over the 6-month trial period. These nine children were sick and absent during the follow-ups. Complete datasets were available for 99 children in the albendazole group and 95 children in the placebo group. No noteworthy difference in baseline socio-demographics or soil-transmitted helminth infection prevalence and intensity was observed between the albendazole and placebo groups (Table 1).

**Table 1:** Baseline characteristics of 194 children from south-west Yunnan province, P.R. China, who participated in a randomized controlled trial conducted from October 2011 to May 2012.

Characteristics^a^	Triple-dose albendazole (n = 99)		Placebo (n = 95)	
	Male (n = 46)	Female (n = 53)	Male (n = 48)	Female (n = 47)
Age [years]	10.4 (1.1)	10.5 (1.2)	10.4 (1.0)	10.2 (1.0)
Illiterate parents	32 (69.6%)	27 (50.9%)	28 (58.3%)	22 (46.8%)
Family relying on farming for income	46 (100%)	53 (100%)	48 (100%)	47 (100%)
**Soil-transmitted helminth prevalence**				
*Ascaris lumbricoides*	46 (100%)	48 (90.6%)	43 (89.6%)	44 (93.6%)
*Trichuris trichiura*	44 (95.7%)	48 (90.6%)	47 (97.9%)	44 (93.6%)
Hookworm	31 (67.4%)	29 (54.7%)	34 (70.8%)	25 (53.2%)
*Taenia* spp.	n.r.	n.r.	1 (2.1%)	n.r.
*Strongyloides stercoralis*	n.r.	2 (3.8%)	1 (2.1%)	3 (6.4%)
**Soil-transmitted helminth infection intensity**				
*Ascaris lumbricoides* [EPG]	17,163 (7,548–59,106)	14,652 (5,922–51,324)	21,579 (4,653–43,425)	21,432 (6,390–65,532)
*Trichuris trichiura* [EPG]	150 (60–690)	222 (72–522)	219 (90–741)	318 (108–738)
Hookworm [EPG]	72 (0–204)	30 (0–132)	48 (0–126)	12 (0–198)
**Physical fitness**				
VO_2_ max estimate [ml kg^−1^ min^−1^]	45.5 (3.0)	44.4 (2.6)	45.7 (3.1)	45.0 (3.3)
20-m laps completed	25.4 (8.9)	22.4 (8.3)	27.2 (9.3)	23.2 (10.1)
**Physical strength**				
Grip strength [kg]	12.9 (3.7)	12.5 (4.5)	13.0 (3.5)	12.1 (3.5)
Standing broad jump distance [cm]	144 (14)	140 (14)	146 (14)	138 (13)
**Anthropometric indicators**				
Body height [cm]	124.8 (6.6)	129.7 (10.0)	125.6 (7.4)	127.0 (6.7)
Body weight [kg]	25.0 (3.8)	27.3 (6.1)	25.5 (4.2)	26.2 (4.7)
Body mass index [BMI; kg m^−2^]	16.0 (1.1)	16.0 (1.4)	16.1 (1.1)	16.1 (1.6)
Wasted^b^	2 (4.4%)	2 (3.8%)	n.r.	3 (6.4%)
Stunted^c^	37 (80.4%)	36 (67.9%)	39 (81.3%)	37 (78.7%)
Sum of skinfolds [mm]	10 (2)	12 (3)	10 (2)	12 (4)
**Hematologic**				
Hemoglobin level [g l^−1^]	159 (19)	162 (28)	159 (24)	155 (32)
Anemic^d^	n.r.	3 (5.7%)	1 (2.1%)	5 (10.6%)

a Values are number of children (%) or mean (standard deviation; SD). For infection intensity, data is presented as median (interquartile range).

b Wasting is defined as ≤−2 BAZ score.

c Stunting is defined as ≤−2 HAZ score.

d Anemia is defined according to WHO age-specific cut-offs: Hb<115 g l^−1^ for ages <12 years; Hb<120 g l^−1^ for ages ≥12 and <15 years.

n.a.: not applicable; n.r.: not represented.

Children were also comparable in terms of physical fitness and strength at baseline. The mean VO_2_ max estimate and number of 20-m laps completed were 44.9 ml kg^−1^ min^−1^ (standard deviation (SD): 2.8 ml kg^−1^ min^−1^) and 23.8 laps (SD: 8.7 laps), respectively, for the albendazole group, and 45.4 ml kg^−1^ min^−1^ (SD: 3.2 ml kg^−1^ min^−1^) and 25.2 laps (SD: 9.9 laps), respectively, for the placebo group. With regard to physical strength, the mean grip strength and standing broad jump distance were 12.6 kg (SD: 4.1 kg) and 142 cm (SD: 14 cm), respectively, for the albendazole group, and 12.6 kg (SD: 3.5 kg) and 142 cm (SD: 14 cm), respectively, for the placebo group. However, when further stratified by sex, boys had higher physical fitness and strength than girls (statistical significance achieved for all indicators except grip strength) (Table 1).

Treatment groups were also comparable in terms of anthropometric and hematologic characteristics at baseline. Despite the mean BMI for the albendazole and placebo group being 16.0 (SD: 1.3) and 16.1 (SD: 1.3) respectively, the baseline prevalence of wasting was only 3.6%. On the other hand, stunting was present in 76.8% (mean height for the albendazole and placebo group was 127.4 cm (SD: 8.9 cm) and 126.3 cm (SD: 7.1 cm), respectively) of the cohort. Baseline prevalence of anemia was low at 4.6%, as the mean hemoglobin levels for the albendazole and placebo group were 161 g l^−1^ (SD: 24 g l^−1^) and 157 g l^−1^ (SD: 28 g l^−1^), respectively.

### Effects of Deworming on Primary and Secondary Outcomes

Children receiving triple-dose albendazole experienced a greater, but mostly not statistically significant, change in the means of their physical fitness scores than their peers from the placebo group at all three follow-ups (Table 2). VO_2_ max estimates increased by 1.0–2.3 ml kg^−1^ min^−1^ from baseline over the 6-month trial period for the albendazole group, while the increase for the placebo group ranged from 0.2–2.1 ml kg^−1^ min^−1^. Likewise for the number of 20-m laps completed, the range of increase was 3.4–11.9 laps and 1.4–11.7 laps for the albendazole and placebo group, respectively. When adjusted for village, and at the individual level for sex, age, height, and weight at follow-up, the difference in the increase of physical fitness between both groups was highest at the 1-month follow-up, where the increase from baseline in the albendazole group was 0.9 ml kg^−1^ min^−1^ (*P* = 0.05) or 2.1 laps (*P* = 0.14) higher than the placebo group. With regard to physical strength, the grip strength increased 0.8–2.0 kg from baseline for the albendazole group, while the placebo group experienced an increase of 0.4–1.8 kg. The difference in the increase between both groups was highest at the 1-month follow-up (0.3 kg higher in the albendazole group), but this difference was not statistically significant. The largest change in standing broad jump distance from baseline was observed among the albendazole group at the 1-month follow-up (+2 cm), but the placebo group fared better at the 6-month follow-up (+2 cm). However, both of these increases were not statistically significant.

**Table 2:** Effects of deworming on changes in physical fitness and strength indicators (primary outcomes) at various follow-ups from baseline among 194 children from south-west Yunnan province, P.R. China, who participated in a randomized controlled trial conducted from October 2011 to May 2012.

	1-month follow-up			4-month follow-up			6-month follow-up		
Characteristics^a^	ALB (n = 99)	PLB (n = 95)	Difference in Δ from baseline^b^	ALB (n = 99)	PLB (n = 95)	Differences in Δ from baseline^b^	ALB (n = 99)	PLB (n = 95)	Differences in Δ from baseline^b^
VO_2_ max estimate [ml kg^−1^ min^−1^]	45.9 (1.0)	45.5 (0.2)	**0.9 (0.0 to 1.8)**	45.7 (0.8)	46.2 (0.8)	0.2 (−0.8 to 1.1)	47.2 (2.3)	47.5 (2.1)	0.2 (−0.7 to 1.2)
20-m laps completed	27.2 (3.4)	26.6 (1.4)	2.1 (−0.7 to 5.0)	29.4 (5.6)	30.2 (5.0)	0.9 (−2.1 to 3.9)	35.7 (11.9)	36.9 (11.7)	0.2 (−2.7 to 3.1)
Grip strength [kg]	13.4 (0.8)	13.0 (0.4)	0.3 (−0.3 to 0.9)	14.5 (1.8)	14.2 (1.6)	0.1 (−0.5 to 0.8)	14.6 (2.0)	14.4 (1.8)	0.1 (−0.6 to 0.9)
Standing broad jump distance [cm]	142 (0)	141 (−1)	2 (−2 to 5)	146 (4)	145 (3)	1 (−2 to 4)	146 (5)	149 (7)	−2 (−5 to 2)

a Values are mean (Δ from baseline), unless otherwise stated.

b Differences in the changes between follow-up and baseline among the intervention groups are adjusted for village, and at the individual level for sex, age at follow-up, and height and weight at baseline (for the 1-month follow-up) or follow-up (for the 4- and 6-month follow-ups). Values are calculated from a multivariate linear regression model, presented as coefficient (95% confidence interval) and highlighted in bold if statistical significance is achieved (*P*<0.05).

ALB: triple-dose albendazole; PLB: placebo.

In terms of secondary outcomes, children in the albendazole group had a larger increase, from baseline, in the means of their body height and weight and sum of skinfolds than their counterparts from the placebo group (Table 3). The range of increase for body height, weight, and sum of skin folds were 2.9–3.5 cm, 1.4–2.2 kg, and 1 mm, respectively, for the albendazole group, and 2.7–3.3 cm, 1.2–1.9 kg, and 1 mm for the placebo group. However, differences between both groups in the change from baseline were statistically non-significant at all follow-ups after adjusting for sex, age at follow-up, and village. A reduction in hemoglobin level was observed in both groups at the 1- and 6-month follow-ups, and the respective reduction in the albendazole group was 2 g l^−1^ (*P* = 0.72) and 3 g l^−1^ (*P* = 0.49) higher compared to the placebo group On the other hand, the increase from baseline in the albendazole group was 3 g l^−1^ higher than the placebo group at the 4-month follow-up (*P* = 0.65).

**Table 3:** Effects of de-worming on changes in nutritional indicators (secondary outcomes) at various follow-ups from baseline among 194 children from south-west Yunnan province, P.R. China, who participated in the randomized controlled trial conducted from October 2011 to May 2012.

	1-month follow-up			4-month follow-up			6-month follow-up		
Characteristics^a^	ALB (n = 99)	PLB (n = 95)	Differences in Δ from baseline^b^	ALB (n = 99)	PLB (n = 95)	Differences in Δ from baseline^b^	ALB (n = 99)	PLB (n = 95)	Differences in Δ from baseline^b^
Body height [cm]	n.d.			130.3 (2.9)	129.0 (2.7)	0.2 (−0.1 to 0.4)	130.9 (3.5)	129.6 (3.3)	0.2 (−0.1 to 0.4)
Body weight [kg]				27.6 (1.4)	27.0 (1.2)	0.2 (−0.1 to 0.4)	28.4 (2.2)	27.7 (1.9)	0.2 (−0.1 to 0.6)
Stunted (%)^c^	n.d.			63 (−10.1%)	70 (−6.3%)	n.a.^d^	66 (−7.0%)	69 (−7.4%)	n.a.^d^
Sum of skinfolds [mm]				12 (1)	12 (1)	0 (0 to 1)	12 (1)	12 (1)	0 (0 to 1)
Hemoglobin level [g l^−1^]	151 (−10)	150 (−7)	−2 (−10 to 7)	164 (3)	159 (2)	3 (−8 to 13)	148 (−13)	148 (−9)	−3 (−11 to 6)

a Values are number of children (% change from baseline) or mean (Δ from baseline), unless otherwise stated.

b Differences in the changes between follow-up and baseline among the intervention groups are adjusted for village, and at the individual level for sex and age at follow-up. Values are calculated from a multivariate linear regression model, presented as coefficient (95% confidence interval) and highlighted in bold if statistical significance is achieved (*P*<0.05). For percentage stunted, the χ^2^ test is used and thus, no 95% confidence interval is presented.

c Stunting is defined as ≤−2 HAZ score.

d *P*-value calculated from χ^2^ test comparing % stunted between ALB and PLB for statistical significance.

ALB: triple-dose albendazole; PLB: placebo; n.d.: not determined.

### Effects of Soil-Transmitted Helminth Infections on Primary Outcomes

**Figure 2:**
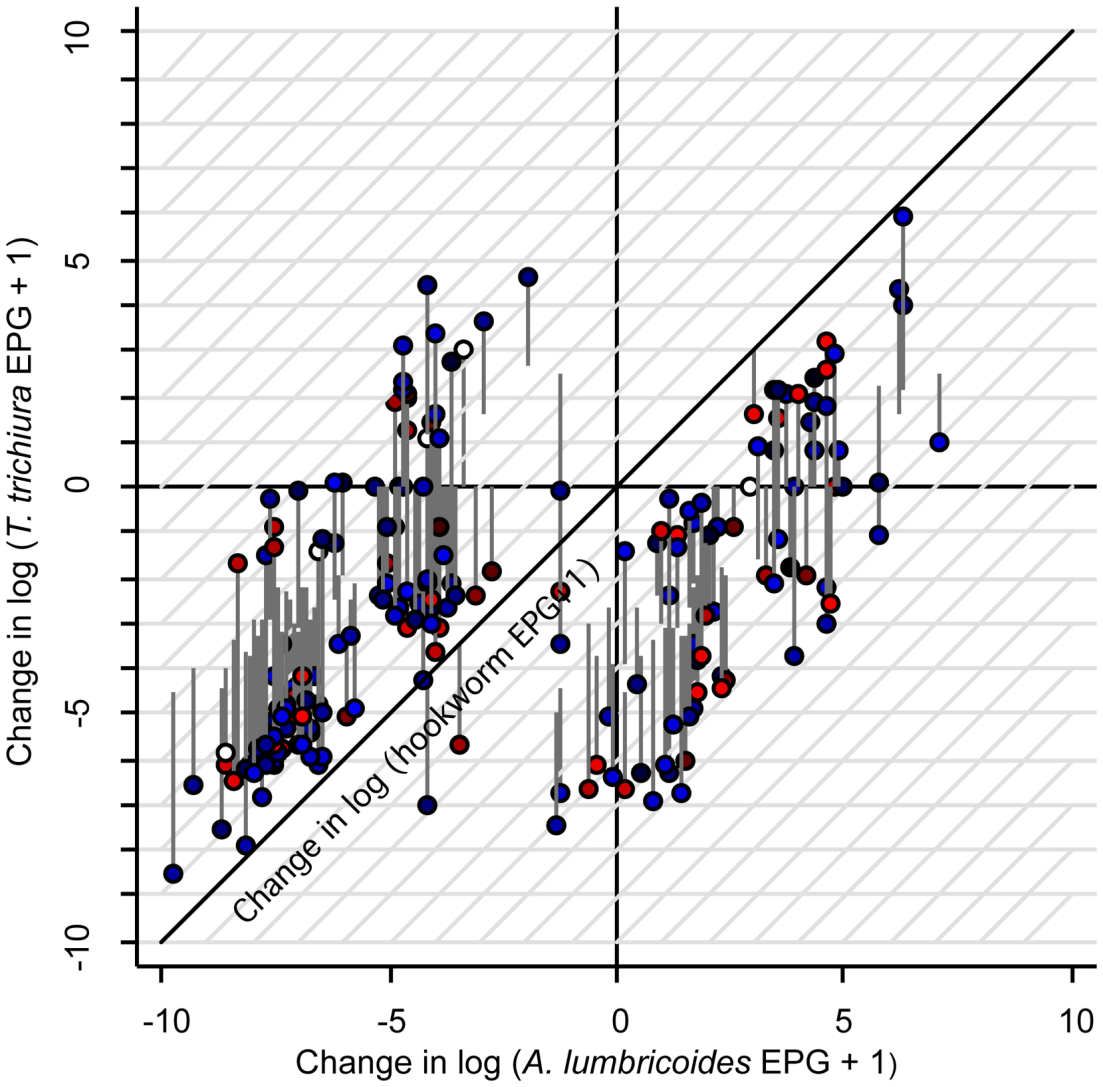
Three-dimensional visualization of changes in 20-m laps due to differences in soil-transmitted helminth infection intensities in Chinese children. The study was carried out in south-west Yunnan province, P.R. China between October 2011 and May 2012 among 194 children aged 9–12 years. The changes in infection intensities between the 4-month follow-up and baseline for *Ascaris lumbricoides*, *Trichuris trichiura*, and hookworm are reflected along the X-, Y-, and Z-axis, respectively. Each circle indicates the change for a particular child harboring a certain mixture of soil-transmitted helminth infection intensities. Blue circles indicate positive change, red circles indicate negative change, and white circles indicate no change. A darker shade of color indicates a greater degree of change.

When the soil-transmitted helminth infection status was used as explanatory variable for the primary outcomes (Table 4), *T. trichiura*-infected children had 1.6 ml kg^−1^ min^−1^ less increase in their VO_2_ max estimate from baseline than their non-infected peers at the 1-month follow-up (*P* = 0.012). Similarly, hookworm-infected children had 1.1 ml kg^−1^ min^−1^ less increase in their VO_2_ max estimate from baseline than their non-infected peers at the 6-month follow-up (*P* = 0.03). In addition, the increase in the number of 20-m laps completed from baseline was 4.6 (*P* = 0.04) and 6.0 (*P* = 0.01) laps less for *T. trichiura*-infected children than their non-infected counterparts at the 1- and 4-month follow-ups, respectively. As further illustrated in Figure 2, an increase from baseline (positive change) in the number of 20-m laps completed at the 4-month follow-up was more dependent on a reduction in *T. trichiura* infection intensity than diminished *A. lumbricoides* and hookworm infection intensity. In terms of grip strength at the 1-month follow-up (Table 4), the increase from baseline among *A. lumbricoides*-infected children was 0.8 kg lower than among children not infected with this helminth species (*P* = 0.05), but hookworm-infected children had 0.9 kg more increase from baseline than their non-infected peers (*P* = 0.04). No statistically significant change in standing broad jump distance due to soil-transmitted helminth infection status was observed at each of the three follow-ups.

**Table 4:** Effects of soil-transmitted helminth infection status on changes in physical fitness and strength indicators (primary outcomes) at various follow-ups from baseline among 194 children from south-west Yunnan province, P.R. China, who participated in the randomized controlled trial conducted from October 2011 to May 2012.

	1-month follow-up		4-month follow-up		6-month follow-up	
Multivariate linear regression models^a^	n	Coefficient (95% CI)	n	Coefficient (95% CI)	n	Coefficient (95% CI)
**(A) Change in VO_2_ max estimate [ml kg^−1^ min^−1^]**						
*Ascaris lumbricoides*-infected	97	−0.1 (−1.2 to 1.0)	167	0.7 (−0.8 to 2.2)	175	1.2 (−0.4 to 2.8)
*Trichuris trichiura*-infected	166	**−1.6 (−3.0 to −0.3)**	170	−1.5 (−3.1 to 0.1)	175	0.5 (−1.1 to 2.1)
Hookworm-infected	59	−0.2 (−1.5 to 1.0)	51	0.4 (−0.8 to 1.6)	56	**−1.1 (−2.1 to −0.1)**
**(B) Change in 20-m intervals completed**						
*Ascaris lumbricoides*-infected	97	−0.2 (−3.7 to 3.4)	167	3.1 (−1.4 to 7.5)	175	3.4 (−1.7 to 8.5)
*Trichuris trichiura*-infected	166	**−4.6 (−8.9 to −0.3)**	170	**−6.0 (−10.7 to −1.2)**	175	1.2 (−3.9 to 6.3)
Hookworm-infected	59	−0.1 (−4.1 to 3.9)	51	2.0 (−1.5 to 5.5)	56	−2.2 (−5.5 to 1.1)
**(C) Change in grip strength [kg]**						
*Ascaris lumbricoides*-infected	97	**−0.8 (−1.5 to 0.0)**	167	−0.5 (−1.5 to 0.5)	175	−1.2 (−2.4 to 0.1)
*Trichuris trichiura*-infected	166	0.0 (−0.9 to 0.8)	170	0.9 (−0.1 to 2.0)	175	0.7 (−0.6 to 2.0)
Hookworm-infected	59	**0.9 (0.1 to 1.7)**	51	0.1 (−0.7 to 0.9)	56	0.7 (−0.1 to 1.5)
**(D) Change in standing broad jump distance [cm]**						
*Ascaris lumbricoides*-infected	97	−2 (−6 to 2)	167	−2 (−7 to 3)	175	1 (−4 to 7)
*Trichuris trichiura*-infected	166	5 (0 to 10)	170	5 (0 to 10)	175	5 (−1 to 11)
Hookworm-infected	59	0 (−4 to 5)	51	−3 (−7 to 1)	56	1 (−3 to 5)

a For each model, the outcome variable is highlighted with a grey bar and the explanatory variables (reference group is always not infected with the particular soil-transmitted helminth species) are presented below. All models have been adjusted for village, and at the individual level for sex, age at follow-up, and height and weight at baseline (for the 1-month follow-up) or follow-up (for the 4- and 6-month follow-ups). Values are presented as coefficient (95% confidence interval) and highlighted in bold if statistical significance is achieved (*P*<0.05).

**Figure 3:**
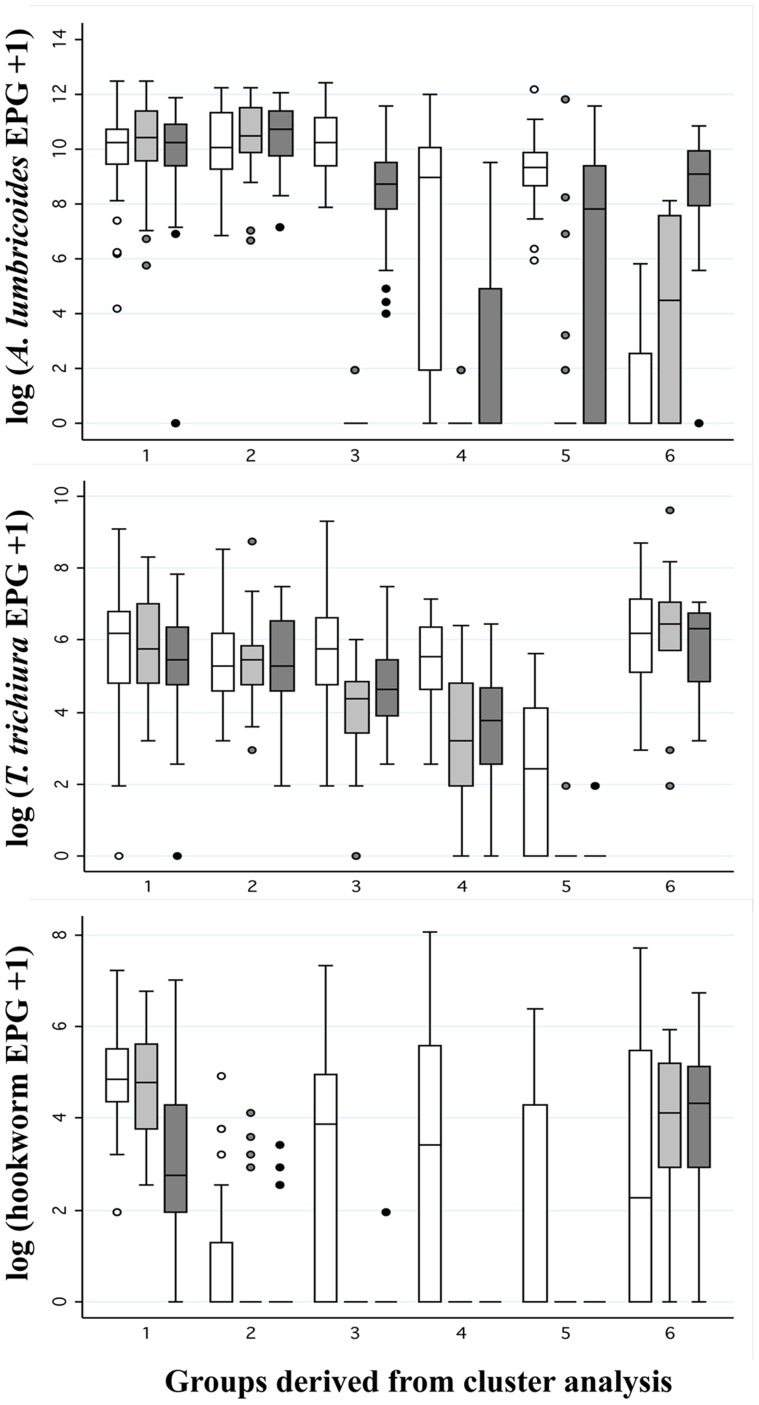
Boxplots of six infection intensity groups identified by principal component and cluster analysis. The groups are based on infection intensities of the three soil-transmitted helminths at baseline (white), 1-month follow-up (light grey), and 4-month follow-up (dark grey), among 194 children from a randomized controlled trial conducted in south-west Yunnan province, P.R. China, from October 2011 to May 2012.

When the children were grouped according to their longitudinal infection intensity patterns, the six groups that emerged (Figure 3) had the following characteristics: group 1, high infection intensity of all species at all time-points; group 2, high infection intensity of all species except hookworm at all time-points; group 3, high intensity of *A. lumbricoides* re-infection by the 4-month follow-up, high infection intensity of *T. trichiura* at all time-points, and no or minimal hookworm re-infection at follow-ups; group 4, low intensity of *A. lumbricoides* re-infection by the 4-month follow-up, intermediate infection intensity of *T. trichiura* at all time-points, and no or minimal hookworm re-infection at follow-ups; group 5, intermediate intensity of *A. lumbricoides* re-infection by the 4-month follow-up, and no or minimal *T. trichiura* and hookworm re-infection at follow-ups; and group 6, infection intensity of all species were higher during the follow-ups compared to the pre-treatment baseline. When group 1 was used as the reference group in the multivariate linear regression models (Table 5), children from group 5 had consistently more increase in their VO_2_ max estimate from baseline than their peers from group 1 at all follow-ups (range: 1.9–2.1 ml kg^−1^ min^−1^; all *P*<0.05). A similar trend was observed for the number of 20-m laps completed and a statistically significant 5.7 more 20-m laps were completed by children from group 5 as compared to group 1 (*P* = 0.04) and the baseline. In terms of standing broad jump distance, children from group 4 had a 6 cm higher increase from baseline than children from group 1 at the 4-month follow-up (*P* = 0.03). No statistically significant change in grip strength dependent on soil-transmitted helminth infection intensity was observed at all follow-ups.

**Table 5:** Effects of soil-transmitted helminth infection intensity on changes in physical fitness and strength indicators (primary outcomes) at various follow-ups from baseline among 194 children from south-west Yunnan province, P.R. China, who participated in a randomized controlled trial conducted from October 2011 to May 2012.

	1-month follow-up	4-month follow-up	6-month follow-up
Multivariate linear regression models^a^	Coefficient (95% CI)	Coefficient (95% CI)	Coefficient (95% CI)
**(A) Change in VO_2_ max estimate [ml kg^−1^ min^−1^]**			
Group 2 (n = 36)	−0.2 (−1.6 to 1.2)	−0.2 (−1.7 to 1.3)	0.7 (−0.7 to 2.1)
Group 3 (n = 53)	0.3 (−0.9 to 1.5)	−0.1 (−1.4 to 1.3)	0.1 (−1.2 to 1.4)
Group 4 (n = 27)	1.2 (−0.3 to 2.7)	0.2 (−1.5 to 1.9)	0.7 (−0.8 to 2.2)
Group 5 (n = 22)	**1.9 (0.3 to 3.5)**	**2.1 (0.3 to 3.9)**	**1.9 (0.2 to 3.6)**
Group 6 (n = 10)	0.3 (−1.9 to 2.4)	0.9 (−1.5 to 3.2)	0.3 (−1.9 to 2.5)
**(B) Change in 20-m intervals completed**			
Group 2 (n = 36)	−1.4 (−5.8 to 3.0)	−2.2 (−6.7 to 2.4)	0.5 (−4.0 to 5.0)
Group 3 (n = 53)	−0.2 (−4.1 to 3.8)	−0.8 (−4.8 to 3.3)	−1.6 (−5.6 to 2.5)
Group 4 (n = 27)	2.5 (−2.3 to 7.3)	0.3 (−4.7 to 5.3)	2.7 (−2.3 to 7.7)
Group 5 (n = 22)	5.1 (0.0 to 10.3)	**5.7 (0.4 to 11.1)**	3.9 (−1.5 to 9.2)
Group 6 (n = 10)	−1.7 (−8.5 to 5.1)	1.4 (−5.6 to 8.5)	0.3 (−6.7 to 7.3)
**(C) Change in grip strength [kg]**			
Group 2 (n = 36)	−0.6 (−1.5 to 0.3)	−0.7 (−1.7 to 0.3)	−1.2 (−2.4 to −0.1)
Group 3 (n = 53)	−0.2 (−1.1 to 0.6)	−0.1 (−1.0 to 0.8)	−0.5 (−1.5 to 0.5)
Group 4 (n = 27)	−0.4 (−1.4 to 0.7)	0.3 (−0.8 to 1.4)	−0.1 (−1.4 to 1.1)
Group 5 (n = 22)	−0.5 (−1.6 to 0.6)	−0.8 (−1.9 to 0.4)	−0.8 (−2.2 to 0.5)
Group 6 (n = 10)	0.0 (−1.5 to 1.4)	0.0 (−1.6 to 1.5)	−0.1 (−2.3 to 1.2)
**(D) Change in standing broad jump distance [cm]**			
Group 2 (n = 36)	−1 (−6 to 4)	1 (−4 to 6)	−2 (−7 to 3)
Group 3 (n = 53)	1 (−4 to 5)	3 (−1 to 7)	−1 (−5 to 4)
Group 4 (n = 27)	4 (−2 to 9)	**6 (1 to 12)**	0 (−5 to 6)
Group 5 (n = 22)	−3 (−9 to 3)	−1 (−7 to 4)	−5 (−11 to 1)
Group 6 (n = 10)	−3 (−11 to 5)	1 (−7 to 9)	1 (−7 to 9)

a For each model, the outcome variable is highlighted with a grey bar and the explanatory variables (reference group is always group 1) are presented below. All models have been adjusted for village, and at the individual level for sex, age at follow-up, and height and weight at baseline (for the 1-month follow-up) or follow-up (for the 4-month follow-up). Values are presented as coefficient (95% confidence interval) and highlighted in bold if statistical significance is achieved (*P*<0.05).

## Discussion

As shown in our preceding work in Bulang communities [24], [26], the prevalence and intensity of soil-transmitted helminth infections in villages inhabited by this ethnic minority group can be very high. For example, in the current randomized controlled trial, we found baseline prevalence of *T. trichiura*, *A. lumbricoides*, and hookworm at 94.5%, 93.3%, and 61.3%, respectively. Therefore, an intensive de-worming regimen, consisting of triple-dose albendazole [24], [33], was employed to allow children a fair chance of developing their physical fitness unaffected by intestinal helminth infections. Re-infection with *A. lumbricoides* occurred more rapidly than expected and the prevalence of *A. lumbricoides* reached 80% of the pre-treatment prevalence 4 months after treatment [24]. Despite triple-dose albendazole treatment, a low cure rate of 19.6% was obtained against *T. trichiura*, corroborating previous conclusions that *T. trichiura* infection is particularly hard to cure with current anthelmintic drugs [16], [34]–[36]. Such re-infection dynamics have complicated the evaluation of the potential health benefits of deworming and rendered the grouping of the children according to intervention near-irrelevant as the treated children might not have benefited from a meaningful helminth-free period for substantial catch-up growth. This finding further suggests that in our study area, the current WHO recommendation of single-dose albendazole (400 mg) twice yearly [15] might be insufficient for controlling soil-transmitted helminthiasis.

In a recently published trial conducted in India [37], where 1 million preschool-aged children, 1 to 6 years old at baseline, were treated with albendazole every 6 months for 5 years, no statistically significant difference in anthropometric measurements was detected between the albendazole and control groups. In our study, even though a trend toward higher values was observed for the treated cohort, no statistically significant difference in most primary and secondary outcomes between the albendazole and placebo groups was detected during the 6-month follow-up period. However, we did find one statistically significant, and biologically important, difference in the VO_2_ max estimate at 1-month follow-up between the albendazole and placebo groups despite the relatively small sample size. It is interesting to note the variation in hemoglobin levels for both groups throughout the follow-up period and this could probably be due to seasonal dietary changes or the presence of other infections. In addition, we observed a general learning effect with the physical fitness and strength tests, especially the 20-m shuttle run test, amongst the children, but this was mitigated in the analysis by having a control group.

In the sub-group analysis, we found that soil-transmitted helminth-infected children had performed significantly worse in the physical fitness and strength tests than their non-infected peers. When we grouped children according to their infection status at each follow-up, we observed that *T. trichiura*-infected children performed worse in the 20-m shuttle run than their non-infected peers. This confirmed the results from a cross-sectional study conducted by our group where *T. trichiura*-infected children were found to complete, on average, 6.1 20-m laps less and have a VO_2_ max estimate which was 1.9 ml kg^−1^ min^−1^ lower than their non-infected counterparts [22]. To survive in a host, adult *T. trichiura* worms anchor their whip-like anterior end into the wall of the large intestine and caecum by secreting pore-forming proteins. Such an invasive mechanism causes inflammation and bleeding, resulting in abdominal pain in the short term, and anemia and rectal prolapse in the long term, especially when large numbers of worms are present [38]. A significant change in physical fitness already at the 1-month follow-up could indicate that removing abdominal pain alone through the expulsion of *T. trichiura* might enhance the host's endurance in exhaustive exercises, such as the 20-m shuttle run. Hookworm-infected children were also found to have a significantly lower increase from baseline in their VO_2_ max estimates than children not infected with hookworm at the 6-month follow-up. Although anemia is a known symptom of hookworm infection and would be a plausible cause for reduced VO_2_ max estimates [21], it was detected in only 10.7% of the hookworm-infected children and no significant association was found between any soil-transmitted helminth infection and hemoglobin level. The migration of the hookworm larvae through the pulmonary blood vessels, where they bore into the alveoli, could offer an alternative explanation to this observation. Although the larvae of *A. lumbricoides* undergo a similar migratory process [38], no reduction in VO_2_ max estimates was observed in children infected with *A. lumbricoides*. In terms of grip strength, the increase from baseline among *A. lumbricoides*-infected children was significantly lower, while hookworm-infected children had a higher increase from baseline, when compared to their non-infected peers. As there is currently limited evidence on the association of soil-transmitted helminth infection and grip strength, these inconsistent findings warrant further investigation.

When children were grouped according to infection intensity, we were able to take into consideration the degree of infection at baseline, 1-month, and 4-month follow-ups, and the extent of multiparasitism for each child. These analyses revealed that individuals with a combination of no or minimal *T. trichiura* and hookworm re-infection achieved higher improvements during the follow-ups in the 20-m shuttle run, as compared to peers with high infection intensity of all species. In addition, children with no or minimal *A. lumbricoides* and hookworm re-infection performed better in the standing broad jump than their counterparts with high infection intensity of all species. These findings provide further evidence of the impact of soil-transmitted helminth infections on the physical fitness and strength of school-aged children.

The anthropometric and physical strength findings from this trial should be viewed in the light of the following limitations. A follow-up period of 6 months is probably too short for an accurate evaluation of anthropometric gains and physical strength increments from longer-term physical growth due to deworming. Taking into account that keeping controls untreated for a long period would be difficult due to ethical considerations, a 3- to 5-year prospective cohort study, where children are treated regularly to ensure that they are helminth-free, and the changes in anthropometric indicators and physical strength from baseline are monitored and compared with changes in soil-transmitted helminth infection intensity over time, could be a more appropriate study design. Finally, catch-up growth after anthelmintic treatment can only occur if the diet is sufficient [39]. Based on the investigators' observations in the field, most of the children's diet consists mainly of white rice with little protein sources. Dietary improvements, in addition to deworming, are therefore necessary in the current setting for catch-up growth to occur and perhaps to aid in the absorption of albendazole, and should be considered in future, more comprehensive studies.

We conclude that there is no strong evidence for significant improvements in physical fitness and anthropometric indicators due to deworming with triple-dose albendazole. This might be partly explained by the rapid re-infection observed with *A. lumbricoides* and low cure rates with *T. trichiura*. However, negative impacts on the physical fitness and strength were observed in school-aged children infected with soil-transmitted helminths in sub-group analyses. In particular, the clear effects of *T. trichiura* infection on physical fitness in this trial is intriguing as the public health burden of this helminth species is currently not as well defined as that of the other two species. The fact that *T. trichiura* infection had the strongest negative impact on the physical fitness of the children but was hardly cured with triple-dose albendazole is another major concern. Finally, we also showed that the observed morbidities were infection intensity-dependent and in order to control them, regular deworming, coupled with dietary improvements and water, sanitation, and hygiene development, should be considered.

## Supporting Information

Checklist S1 **CONSORT checklist**
(DOC)

Protocol S1 **Study protocol for trial entitled “Effect of deworming on physical fitness of school-aged children in Yunnan, China: a double-blind, randomized, placebo-controlled trial.”**
(DOC)
