# Supplementary material for: Effect of Deworming on Physical Fitness of School-Aged Children in Yunnan, China: A Double-Blind, Randomized, Placebo-Controlled Trial
Source: PLoS Negl Trop Dis. 2014 Jul 10;8(7):e2983. doi: 10.1371/journal.pntd.0002983 (PMC4091871; doi:10.1371/journal.pntd.0002983)
Supplement: Protocol S1 — Study protocol for trial entitled “Effect of deworming on physical fitness of school-aged children in Yunnan, China: a double-blind, randomized, placebo-controlled trial.” (DOC) [file pntd.0002983.s002.doc]

**Version 2 (30.9.2011)**

**Effect of deworming on physical fitness of school-aged children in Yunnan, China: a double-blind, randomized, placebo-controlled trial**

**Study Protocol**

Prepared by

Peiling Yap

Supervision

Dr. Peter Steinmann

Department of Epidemiology and Public Health
Swiss Tropical and Public Health Institute, Basel, Switzerland

Prof. Dr. Jürg Utzinger

Department of Epidemiology and Public Health

Swiss Tropical and Public Health Institute, Basel, Switzerland

**1. Study design**


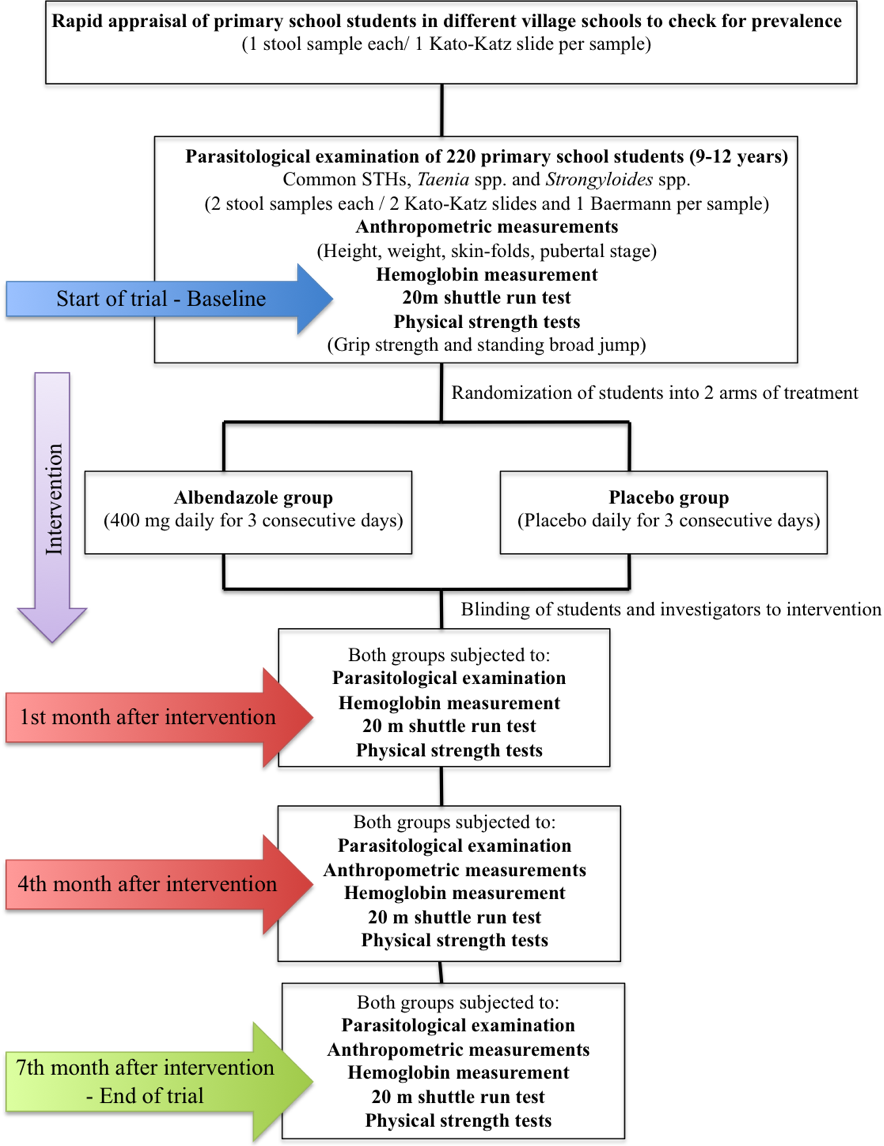


**1.1 Rapid appraisal**

6 village schools in Zhangjia have been identified, based on willingness of villagers to participate and ease of transporting stool samples to a local makeshift laboratory, as potential schools for the trial. A rapid appraisal will be conducted during the first week of the field-work and depending on the number of primary school children present in each village school, 10-15 children, aged 9-12 years, will be randomly selected and asked to provide 1 stool sample for parasitological examination with the Kato-Katz method (1 slide per sample). Only village schools with a prevalence of 70% or more in any of the common soil-transmitted helminths (STHs; *Ascaris lumbricoides, Trichuris trichiura* and hookworm) will be included for the recruitment process of the trial.

**1.2 Recruitment, baseline, intervention and follow-ups**

*Recruitment* - Given an estimated 70% prevalence and 50% loss to follow-up, **250** children have to be recruited to achieve a power of 80% at an alpha error of 5% to detect statistically significant differences in the physical fitness (difference of 2.5 mL/kg/min is considered to be of clinical relevance) and strength values between the albendazole and placebo groups. In each village, the village doctor, chief and teachers will be briefed on the aims of the study. All primary school children, aged 9-12 years will be gathered and the teachers will explain the study to the children and encourage them to participate in it, highlighting the physical fitness and strength tests at the end of the parasitological examination. Written informed consent will be signed by the childrens’ parents/guardians on their behalf.

*Baseline* - Once informed consent has been obtained, the 1^st^ labeled (name, ID number) stool container will be distributed and collected the next day. During the collection of the 1^st^ stool container, the 2^nd^ labeled stool container will be given out and collected the day after. Only children who have submitted 2 stool samples and are infected with one or more of the common STHs will be included in further tests and follow-ups. All infected children are subjected to anthropometric measurements, hemoglobin measurements, 20 m shuttle run test, grip strength test and standing broad jump test.

*Intervention* – Only infected children who have completed all the baseline assessments will be randomly allocated to the two treatment arms:

Treatment arm 1: triple-dose albendazole (400 mg daily for 3 consecutive days)

Treatment arm 2: triple-dose placebo (daily for 3 consecutive days)

Each dose of treatment will be provided in person everyday for 3 consecutive days. During the administration of the treatments, children will have to swallow the tablets on the spot and in front of a field member. Water will be provided.

*Follow-ups* – The 1^st^, 2^nd^ and 3^rd^ follow-ups will be performed in December 2011, March and June 2012 respectively. In the 1^st^ follow-up, only the parasitological examination, hemoglobin measurements, 20 m shuttle run test, grip strength test and standing broad jump test will be carried out. While in the 2^nd^ and 3^rd^ follow-ups, all the examination and tests as performed at baseline will be repeated.

*End of trial* – All participants will be offered a free single dose of 400 mg albendazole.

**1.3 Randomization and blinding procedures**

Block randomization will be carried out to ensure that both treatment arms have roughly equal sample sizes. Jan (Hattendorf) will hold the code to the randomization. In the field, class lists from the schools will be obtained and following the order of the list, each child will be assigned a random number from a list of 250 random numbers generated from R [“sample (1:250, 250)”]. This random number will be the number on the packed treatment to be given to the child.

This trial will be double blinded. The albendazole and placebo tablets will look similar and the children will not be told about the presence of the placebo tablets. All investigators in the field will also be blinded to the treatment status of the children, since Steffi (Knopp) will carry out the packing of the tablets in Basel. For each child, the three doses of tablets (albendazole or placebo) will be wrapped with a piece of aluminium foil and sealed in a small Ziploc bag and labeled with a number (from 1-250).

**2. Inclusion and exclusion criteria**

**2.1 Participants inclusion criteria**

1. Aged 9-12 years, male or female
2. Written informed consent by a parent/guardian on behalf of the child
3. Submission of 2 stool samples at baseline
4. Completion of anthropometric and haemoglobin measurements at baseline
5. Completion of 20 m shuttle run test at baseline
6. Completion of grip strength test and standing broad jump test at baseline
7. Infected with one or more common soil-transmitted helminths (*Ascaris lumbricoides, Trichuris trichiura*, hookworm)
8. Absence of major systemic illnesses, as assessed by a medical doctor at baseline
9. No known or reported drug allergy to albendazole
10. Anticipated residence in the study area for at least 1 year

**2.2 Participants exclusion criteria:**

1. Children below the age of 9 years or above 12 years
2. No written informed consent
3. Less than 2 stool samples submitted at baseline
4. Presence of medical condition that prevents child from completing the physical fitness and strength tests or refusal of child to do the tests.
5. No infection with any soil-transmitted helminth.
6. Presence of systemic illness, as assessed by a medical doctor at baseline,
7. Known or reported drug allergy to albendazole
8. Attending other clinical trials during the study period
9. Received anthelminthics in the previous 6 months

**3. Field and laboratory procedures:**

**3.1 Parasitological examination**

Stool samples will be collected and analyzed on the same day. For each of the 2 stool samples collected from each child, **2 Kato-Katz slides and 1 Baermann** will be carried out. Each stool sample will first be visually inspected for *Taenia* spp. proglottids before further examinations take place. In the Kato-Katz test, the 41.7 mg plastic template is used to prepare the fecal thick smears. Slides are read with a light microscope, 60 minutes after they are prepared, for the eggs of *A. lumbricoides, T. trichiura* and hookworm. In the Baermann technique, a large amount of stool sample is placed on a medical gauze in a glass funnel filled with tap water. The whole set up is treated with artificial light directed at the bottom of the funnel for 2 hours. After which, the lowest portion of the liquid (50 mL) is collected, centrifuged (2000 rpm for 5 minutes), the supernatant gently poured off and the sediment subjected to microscopic examination for the larvae of *Strongyloides stercoralis*. Taking the presence of *Taenia* spp. and *S. stercoralis* into account will allow the control of potential confounders in the form of additional intestinal helminth infections.

**3.2 Anthropometric measurements**

(I) Body weight – Each child will be asked to take off his/her shoes and sweater before standing on the digital weighing scale. Weight will be measured (to the nearest 0.1 kg) **twice and averaged**.

(II) Height – With the shoes off, each child will stand against a stadiometer with their back erect but shoulders relaxed. Height will be taken (to the nearest 0.1 cm) **twice and averaged**.

(III) Body mass index (BMI) and 2 Z scores will be calculated:

**BMI** = weight (kg) / (standing height [meters (m)]^2^)

BMI-for-age (**BMIZ**: an indicator for weight-for-height proportion; WHO growth charts)

Height-for-age (**HAZ**: an indicator for chronic nutritional status; WHO growth charts)

(IV) The thickness of the skinfold will be measured at 2 sites, namely **triceps and subscapular** (refer to Figure 1). Before the measurement begins, the field worker will show the Holtain skinfold caliper to the child and clamp it normally on the child’s finger to show that the process will not hurt. During the measurement, the child will stand with arms and shoulders relaxed. The field worker will gently pinch the skin, with the thumb and forefinger, slightly above the middle of the back of the arm so that the skinfold is running vertically and clip the caliper (mouth of caliper should be perpendicular to the skinfold) onto the skinfold in the middle of the back of the arm (triceps). After counting for 4 seconds, the reading should stabilize and be recorded. Release the pinch but let the fingers stay in the same position on the arm and repeat the measurements for another 2 times. The 3 values obtained should be about 5% ± from each other. If this is not the case, repeat the measurements again. The final reading will be **an average of the 3 values**. The same procedures apply for the subscapular site. Skinfold to measure will be directly underneath the shoulder blade.


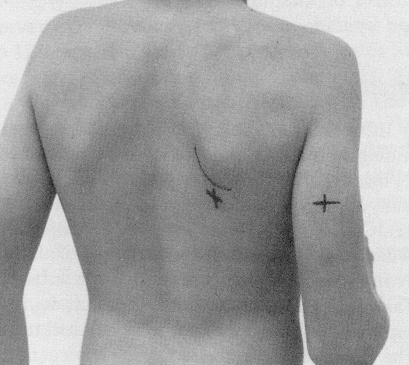


Figure 1 Sites for skinfold measurements

**3.3 Hemoglobin measurement**

The hemoglobin concentration will be **measured once** (to the nearest 0.1 g/L) with the HemoCue® Hb 301 system. A fresh set of alcohol swab, safety lancet and microcuvette will be used for each child. After swabbing the ear lobe with alcohol, the field worker will prick it with a safety lancet and squeeze the ear lobe gently for two drops of blood. The first drop will be wiped away with the alcohol swab and the **second drop will be taken up** by the microcuvette and read by the machine.

**3.4 20 m shuttle run test**

The 20 m flat running course will be measured with a measuring tape and marked with cones. In particular, 5 running lanes will be created. Before the start of the test, all the children will be told to voice out any body discomfort and anyone who feels sick or not comfortable will not take part in the test. The pre-recorded sound signals will be played to the children and they will get to do a trial run of 2 intervals (40 m). Once they are familiar with the test, they will be asked to run, in groups of five, back and forth on the 20 m flat course by following the pace of sound signals. Starting with a running speed of 8.5 km/h, the frequency of the signal increases gradually such that every minute, the pace increases by 0.5 km/h. When a child fails to follow the pace in two consecutive intervals, he/she will be asked to stop and the **stage and the distance completed fully will be recorded**. The age of the participating child and the speed at which the child stopped running will be converted into VO_2_ max estimates using the equation suggested by Leger et al.

**3.5 Grip strength test**

The TKK dynamometer will be used for this test. Before the start of the test, the hand span (distance from the tip of the thumb to the tip of the little finger) of the child’s dominant hand will be measured (to the nearest 0.5 cm) and the grip span on the dynamometer adjusted according to the values in Figure 2. The field worker will also demonstrate how to grip the dynamometer to the child. The child should stand straight, yet relaxed and grip the dynamometer with the arm fully extended. During this time, no other parts of the body should touch the dynamometer. Each child will then have **2 tries** (with a 15-seconds rest in between) to grip the dynamometer as hard as possible with their dominant hand and the **maximum reading** (measured to the nearest 0.1 kg) will be recorded.


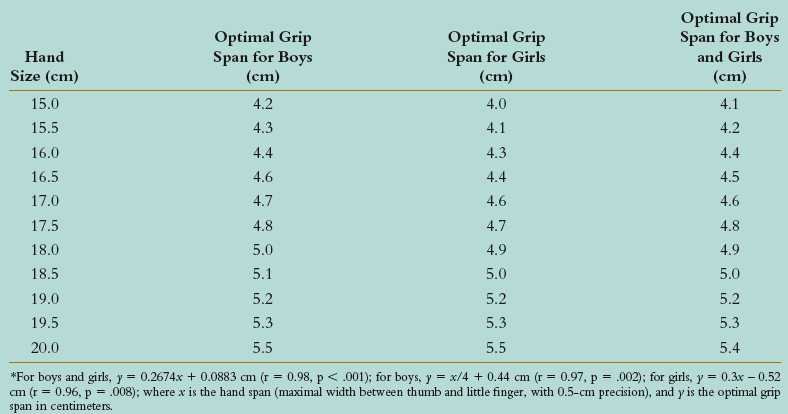


Figure 2 Optimal grip span for each hand span

**3.6 Standing broad jump test**

Before the start of the test, the field worker will demonstrate how to do the standing broad jump. Each child will then stand behind a straight line and jump as far forward as possible with both legs. He/she will have **2 tries** (with a 15-seconds rest in between) and the **longest jump** will be recorded (to the nearest 0.1 cm). The distance of the jump will be measured from the starting line to the heel of the most back foot.

For components 2.2-2.6, **3 stations will be set up, each attended by 2-3 field workers**. The 1^st^ station will consist of the anthropometric and hemoglobin measurements. The 2^nd^ station will consist of the grip strength and standing broad jump tests and the 3^rd^ station will consist of the 20 m shuttle run test. Each child will hold on to a record sheet and as they move from one station to the other, the results of the various measurements and tests will be recorded on it. Once he/she has completed all the stations, the record sheet will be collected. During all the physical fitness and strength tests, the village doctor will be present to ensure that medical attention could be given immediately if required. All measurements and tests will take place from 9am-12pm in the morning and 2-5pm in the afternoon. The order and timing at which the different village schools will be approached will be kept approximately the same at baseline and the subsequent follow-ups. **Air temperature and humidity** of the testing areas will also be monitored.

**4. Data entry and analysis**

The collected data will be double-entered into Excel, mismatches double-checked against the paper copies, and all data merged into a single database for statistical analysis with STATA (version 10.0).

At the end of the trial, the code will be broken and the data analyzed in an un-blinded manner. Parasitological status will be assessed in terms of infection intensity (mean EPG) of individual intestinal helminth species, cure and re-infection rates. Anthropometric measurements, fitness scores and hemoglobin concentrations will be expressed as means and compared between the different intervention groups.

5. Timeline (in detail from Oct – Dec 2011)

| **Date 日期** | | **Activities 活动** |
| --- | --- | --- |
| **Oct 2011十月** | |  |
| 4.10-7.10 | | Arrival in Shanghai on the 5th; Meeting with Prof Zhou on the 7th. 履行至上海；会见周所长 |
| 8.10 | | Travel to Simao;. 履行至思茅 |
| 9.10 | | Check through lab and field materials prepared by Dr Du and team for the trial; Travel to Menghai. 检查全部实验室和现场工作所需物品；履行至勐海。 |
| 10.10 | | Briefing of field team of key aspects of trial; Prepare informed consent and laboratory record forms; Travel to Zhangjia (6 villages); Setting up of laboratory and prepare materials for the parasitological examination (rapid appraisal, 选点); Distribution of stool containers (15 per village) for 2 villages (三队，老寨)。履行至布朗山（章家6村）整理实验室、准备寄生虫检查物品；发粪盒（每村15个）给两个村（三队，老寨）。 |
| 11.10 | | Collection of stool samples; Parasitological examination (1 stool sample per person; 1 Kato-Katz slide per sample); Distribution of stool containers for 2 villages (四队，新囡)。收集粪样。寄生虫检查（一粪一检）。 |
| 12.10 | | Collection of stool samples; Parasitological examination; Distribution of stool containers for 2 villages (空坎1队， 空坎2队)。收集粪样；寄生虫检查；发粪盒给两个村（空坎1队，空坎2队）。 |
| 13.10 | | Collection of stool samples; Parasitological examination; Distribution of stool containers for 2 villages in Xinlong (曼别，曼纳)。收集粪样；寄生虫检查；到新龙的曼别和曼纳发粪盒.?? |
| 14.10 | | Collection of stool samples; Parasitological examination. 收集粪样；检查。 |
| 15.10-16.10 | | Depending on the prevalence obtained from the above 8 villages, if they all fit the criteria of more than 70% for any of the common STHs, they will be selected for the trial and the baseline parasitological examination will start. Otherwise, more villages will be sampled during this time. 根据上述8个村得到的感染率情况，土源性线虫感染率大于70%的村将被选作试验点，开始进行基线寄生虫学调查。若达不到将继续在其它村选择。 |
| 17.10 | | Briefing of village head, doctor and teachers; Recruitment (with informed consent); Distribution of 1^st^ stool containers for 三队，老寨 during school hours (Estimated 102 students aged 9-12 years); Testing of dynamometer (grip span). 向村长、医生和老师简单介绍；发知情同意书；在学生在校期间给三队和老寨的学生发第一次粪盒（估计有102个9～12岁的学生）。（研究正式开始， 粪样检测） |
| 18.10 | | Collection of 1^st^ stool samples; Distribution of 2^nd^ stool containers for 三队，老寨 during school hours; Parasitological examination (2 Kato-Katz slides and 1 Baermann per sample).收集粪样，收到第一次粪样者，同时发给第二次粪盒；寄生虫学检查（改良加滕一粪2检，贝尔曼一粪一检）。 |
| 19.10 | | Collection of 2^nd^ stool samples; Parasitological examination; Briefing of village head, doctor and teachers; Recruitment (with informed consent); Distribution of 1^st^ stool containers for 四队，新囡 during school hours (Estimated 45 students aged 9-12 years).收集三队和老寨学生第二次粪样，寄生虫检查同上；向村长、医生和老师简单介绍；发知情同意书；在学生在校期间给四队和新囡的学生发第一次粪盒（估计有45个9～12岁的学生）。 |
| 20.10 | | Collection of 1^st^ stool samples; Distribution of 2^nd^ stool containers for 四队，新囡 during school hours; Parasitological examination. 收集第一次粪样，收到第一次粪样者，同时发给第二次粪盒；寄生虫检查同上。 |
| 21.10 | | Collection of 2^nd^ stool samples; Parasitological examination. |
| 22.10-23.10 | | Break on weekend. 周末度假。 |
| 24.10 | | Briefing of village head, doctor and teachers; Recruitment (with informed consent); Distribution of 1^st^ stool containers for 空坎1队， 空坎2队 during school hours (Estimated 59 students aged 9-12 years). 收集四队和新囡村学生第二次粪样，寄生虫检查同上；向村长、医生和老师简单介绍；补知情同意书；在学生在校期间给空坎1队和空坎2队的学生发第一次粪盒（估计有59个9～12岁的学生）。 |
| 25.10 | | Collection of 1^st^ stool samples; Distribution of 2^nd^ stool containers for 空坎1队， 空坎2队 during school hours; Parasitological examination. 收集粪样，收到第一次粪样者，同时发给第二次粪盒；寄生虫检查同上。 |
| 26.10 | | Collection of 2^nd^ stool samples; Parasitological examination; Briefing of village head, doctor and teachers; Recruitment (with informed consent); Distribution of 1^st^ stool containers for 曼别，曼纳 during school hours (Estimated 68 students aged 9-12 years). 收集空坎1队和空坎2队村学生第二次粪样，寄生虫检查同上；向村长、医生和老师简单介绍；补知情同意书；在学生在校期间给曼别和曼纳的学生发第一次粪盒（估计有68个9～12岁的学生）。 |
| 27.10 | | Collection of 1^st^ stool samples; Distribution of 2^nd^ stool containers for 曼别，曼纳 during school hours; Parasitological examination. 收集粪样，收到第一次粪样者，同时发给第二次粪盒；寄生虫检查同上。 |
| 28.11 | | Collection of 2^nd^ stool samples; Parasitological examination. 收集第二次粪样，寄生虫检查同上。 |
| 29.10-30.10 | | Training of field workers for anthropometric measurements and physical fitness. |
| **Nov 2011** | |  |
| 31.10 | | Anthropometric measurements, physical fitness tests and hemoglobin measurement (Baseline for 三队); (开始测体能，血红蛋白，身高体重) |
| 1.11 | | Anthropometric measurements, physical fitness tests and hemoglobin measurement (Baseline for 老寨，空坎2队). |
| 2.11 | | Anthropometric measurements, physical fitness tests and hemoglobin measurement (Baseline for 四队，新囡). |
| 3.11 | | Anthropometric measurements, physical fitness tests and hemoglobin measurement (Baseline for 空坎1队). |
| 4.11 | | Anthropometric measurements, physical fitness tests and hemoglobin measurement (Baseline for 曼别，曼纳). |
| 5.11- 6.11 | | Break from weekend 周末度假 |
| 7.11 | | Start of intervention (发药)for all villages (1^st^ dose) 第一次发药。 |
| 8.11 | | 2^nd^ dose for all villages第二次发药。 |
| 9.11 | | 3^rd^ dose for all villages第三次发药 |
| 10.11-11.11 | | Extra days for intervention if needed. (发药) |
| 12.11-14.11 | | Testing of nutritional assessment methods in Upper Nanwen village (3-5 families). 南温上寨营养观测。 |
| 15.11-27.11 | | Break from fieldwork; Data entry. |
| **Dec 2011十二月** | |  |
| 28.11 | | 1^st^ follow up starts; Distribution of 1^st^ stool containers for 三队，老寨 during school hours (Estimated 102 students aged 9-12 years). （粪样检测）开始第一次随访，发粪盒给三队和老寨的在校学生（估计102个9～12的学生）。 |
| 29.11 | | Collection of 1^st^ stool samples; Distribution of 2^nd^ stool containers for 三队，老寨 during school hours; Parasitological examination (2 Kato-Katz slides and 1 Baermann per sample). 收第一次粪样，收到第一次粪样者同时发给第二次粪盒。寄生虫学检查同上。 |
| 30.11 | | Collection of 2^nd^ stool samples; Parasitological examination; Distribution of 1^st^ stool containers for 四队，新囡 during school hours (Estimated 45 students aged 9-12 years).收集第二次粪样。给四队和新囡的在校学生发第一次粪盒（估计有45个9～12岁的学生）。 |
| 1.12 | | Collection of 1^st^ stool samples; Distribution of 2^nd^ stool containers for 四队，新囡 during school hours; Parasitological examination. 收第一次粪样，收到第一次粪样者同时发给第二次粪盒。寄生虫学检查同上。 |
| 2.12 | | Collection of 2^nd^ stool samples; Parasitological examination. |
| 3.12-4.12 | | Break from weekend. |
| 5.12 | | Distribution of 1^st^ stool containers for 空坎1队， 空坎2队 during school hours (Estimated 59 students aged 9-12 years). 收集第二次粪样。给空坎1队和空坎2队的在校学生发第一次粪盒（估计有59个9～12岁的学生）； |
| 6.12 | | Collection of 1^st^ stool samples; Distribution of 2^nd^ stool containers for 空坎1队， 空坎2队 during school hours; Parasitological examination. 收第一次粪样，收到第一次粪样者同时发给第二次粪盒。寄生虫学检查同上。 |
| 7.12 | | Collection of 2^nd^ stool samples; Parasitological examination; Distribution of 1^st^ stool containers for 曼别，曼纳 during school hours (Estimated 68 students aged 9-12 years). 收集第二次粪样。给曼别和曼纳的在校学生发第一次粪盒（估计有68个9～12岁的学生）。 |
| 8.12 | | Collection of 1^st^ stool samples; Distribution of 2^nd^ stool containers for 曼别，曼纳 during school hours; Parasitological examination. 收第一次粪样，收到第一次粪样者同时发给第二次粪盒。寄生虫学检查同上。 |
| 9.12 | | Collection of 2^nd^ stool samples; Parasitological examination. 收第一次粪样，寄生虫学检查同上。 |
| 10.12-11.12 | | Training of field workers for anthropometric measurements and physical fitness. |
| 12.12 | | Physical fitness tests and hemoglobin measurement (1^st^ follow up for 三队); (测体能，血红蛋白) |
| 13.12 | | Physical fitness tests and hemoglobin measurement (1^st^ follow up for 老寨，空坎2队). |
| 14.12 | | Physical fitness tests and hemoglobin measurement (1^st^ follow up for 四队，新囡). |
| 15.12 | | Physical fitness tests and hemoglobin measurement (1^st^ follow up for 空坎1队). |
| 16.12 | | Physical fitness tests and hemoglobin measurement (1^st^ follow up for 曼别，曼纳). |
| 17.12 | | Travel back to Shanghai. 履行至上海 |
| 18.12 | | Travel back to Switzerland. 履行至瑞士 |
| **Mar 2012三月** | |  |
| 5.3-16.3 | | Parasitological examination (2^nd^ follow-up). 寄生虫病检测（第二次随访） |
| 19.3 – 23.3 | | Physical fitness tests, anthropometric measurements and hemoglobin measurement (2^nd^ follow-up).体能、血红蛋白测试（第二次随访） |
| **Jun 2012 六月** | |  |
| 7.6 - 18.6 | | Parasitological examination (3^rd^ follow-up). 寄生虫病检测（第三次随访） |
| 21.6 – 25.6 | | Anthropometric measurements, physical fitness tests and hemoglobin measurement (3^rd^ follow-up). 体能、血红蛋白测试（第三次随访） |
|  | |  |
|  | Fieldwork | |
